# Screening Method for Anti-Colon Cancer Drugs Using Two Sensor Cell Lines with Human β4-Galactosyltransferase 4 Gene Promoters

**DOI:** 10.3390/s18082573

**Published:** 2018-08-06

**Authors:** Naomichi Fukushima, Atena Sugiyama, Takeshi Sato

**Affiliations:** Laboratory of Glycobiology, Department of Bioengineering, Nagaoka University of Technology, Nagaoka, Niigata 940-2188, Japan; naomichi0019@gmail.com (N.F.); yuppi921@aol.com (A.S.)

**Keywords:** β4-galactosyltransferase 4, transcriptional mechanism, sensor cells, colon cancer, drug screening

## Abstract

The increased expression of β4-galactosyltransferase (β4GalT) 4 is closely associated with poor prognosis of colon cancer. Recently, we showed that the expression of the β4GalT4 gene is regulated by the 0.17 kb core promoter region containing one binding site for Specificity protein 1 (Sp1). To develop a screening method for anti-colon cancer drugs, two sensor cell lines having the luciferase gene under the control of two β4GalT4 gene promoters that differed in length were established from SW480 human colon cancer cells. The hGT4-0.17-sensor cells possessed the luciferase reporter driven by the 0.17 kb promoter, while the hGT4-0.3-sensor cells possessed the luciferase reporter driven by the 0.3 kb promoter containing one binding site each for colon cancer-related transcription factors including activator protein 2, E2F, caudal-related homeobox transcription factors, and Runt-related transcription factors besides Sp1. Upon treatment with mitogen-activated protein kinase signaling inhibitor U0126, the promoter activities of the hGT4-0.3-sensor cells decreased significantly, while those of the hGT4-0.17-sensor cells remained unchanged. These results suggest that the responsiveness to U0126 differs between two sensor cell lines due to the different regulation of the luciferase reporters. This study provides the screening method for anti-colon cancer drugs by the combination of two sensor cell lines.

## 1. Introduction

Upon malignant transformation, cell surface glycosylation changes drastically [1,2]. The changes of glycosylation attribute to the modulation of function of cell adhesion molecules and receptors [3,4]. By changing the cell surface glycosylation upon treatment with the inhibitors for glycan biosynthetic pathway such as castanospermine and swainsonine, the metastatic potentials of cancer cells have been shown to decrease [5,6]. However, such inhibitors are not specific to the cancer cell types. Therefore, the inhibitors specific to the cancer cell types need to be discovered.

Galactose (Gal) is a constitutive monosaccharide of glycans. The Galβ1→4 structures are commonly found and synthesized by β4-galactosyltransferase (β4GalT). There are seven members in the β4GalT family [7]. Previously, we showed that by regulating the expression level of the β4GalT5 gene, the malignant properties of cancer cells are suppressed [8]. The findings suggest that if the inhibitors for the expression of the β4GalT genes, which relate to the malignant potentials of specific cancer cell types, are discovered, the inhibitors are useful for cancer therapy. Among the β4GalT family members, the clinical relevance of β4GalT4 was reported that the expression of β4GalT4 increases in colon cancer, and enhanced expression of β4GalT4 is associated with metastasis and poor prognosis of colon cancer [9]. Therefore, β4GalT4 is considered to be a potential target molecule for anti-colon cancer drugs. Recently, we identified the core promoter region of the β4GalT4 gene and showed that the Specificity protein 1 (Sp1)-binding site (−88/−76) in the 0.17 kb core promoter region is critical for the promoter activity in SW480 human colon cancer cells [10].

Development of the specific and highly sensitive drug screening system is an important issue for cancer therapy. However, in order to obtain the information about the effects of the inhibitors on the glycan structures, the fine glycan structures are necessary to be determined by the instrumental analyses including high performance liquid chromatography, nuclear magnetic resonance, and mass spectrometry after purifying the glycans [11]. Since it takes too long to analyze the fine glycan structures, such methods are unsuitable for the first screening for drugs. The previous reports showed that the cell-based biosensors using the luciferase gene under the control of the specific gene promoters have great advantages in sensitivity and processing speed [12,13,14,15,16,17,18]. The methods were utilized in the screening for *Mycobacterium tuberculosis* drugs, immunosuppressive drugs, vascular endothelial growth factor inhibitors, anti-human immunodeficiency virus type 1 drugs, and antimalarial drugs [14,15,16,17,18]. Thus, by focusing on the transcriptional mechanism of the β4GalT4 gene, a screening method for anti-colon cancer drugs that inhibits the expression of the β4GalT4 gene can be developed. 

In the present study, we established two sensor cell lines having the luciferase gene under the control of the β4GalT4 gene promoters from SW480 cells, analyzed the responsiveness of the sensor cells to two signal transduction inhibitors as model compounds, and showed the potential usefulness for the screening of anti-colon cancer drugs.

## 2. Materials and Methods

### 2.1. Chemicals

Hygromycin B was obtained from WAKO Pure Chemical Industries, Ltd. (Osaka, Japan). Mithramycin A was purchased from Sigma-Aldrich (St. Louis, MO, USA). Rabbit anti-p44/42 mitogen-activated protein kinase (MAPK) and anti-phospho-p44/42 MAPK (T202Y204) antibodies, LY294002, and U0126 were from Cell Signaling Technology, Inc. (Danvers, MA, USA).

### 2.2. Cell Culture

SW480 cells were obtained from the Institute of Development, Aging and Cancer, Tohoku University, and cultured in Dulbecco’s modified Eagle’s medium (DMEM) containing 10% fetal calf serum (FCS), 50 units/mL penicillin and 50 μg/mL streptomycin. 

### 2.3. Reporter Plasmid Construction

In our previous study, the reporter plasmids, pGL3-0.3 and pGL3-0.17, in which the promoter regions −253/+47 and −122/+47 of the β4GalT4 gene relative to the transcriptional start site were inserted into the firefly luciferase reporter vector, pGL3-Basic (Promega, Madison, WI, USA), were constructed [10]. To establish the stable sensor cells having the luciferase gene under the control of the β4GalT4 gene promoters from SW480 cells, two reporter plasmids containing 0.3 kb and 0.17 kb promoter regions were prepared using pGL4.15[luc2p/Hygro] vector (Promega), which contains hygromycin-resistant gene. In brief, after the KpnI-BglII fragments were excised from pGL3-0.3 and pGL3-0.17, the 0.3 kb and 0.17 kb DNA fragments were inserted between KpnI and BglII sites of pGL4.15[luc2p/Hygro] vector to generate pGL4-0.3 and pGL4-0.17, respectively.

### 2.4. Establishment of Sensor Cell Lines

To establish the hGT4-0.3- and hGT4-0.17-sensor cell lines, the plasmids pGL4-0.3 and pGL4-0.17 (4 μg each) were transfected by electroporation (500 μF and 250 V) into SW480 cells (2.5 × 10^6^ cells in 0.4 cm cuvette) using a Gene Pulser Xcell CE system (Bio-Rad Laboratories Inc., Hercules, CA, USA). Similarly, the plasmid pGL4.15[luc2p/Hygro] was transfected into SW480 cells to establish the control cell line. The plasmid-transfected cells were selected with DMEM containing 5% FCS and hygromycin B (1 mg/mL) for two weeks.

### 2.5. Treatment with Compounds

The control and sensor cells (1 × 10^5^) in DMEM containing 10% FCS were seeded into 35 mm tissue culture dishes, cultured for 24 h, and then treated with 0.1 μM, 1 μM mythramycin A suspended in ethanol or ethanol as a control for 48 h. In the case of the treatment with U0126 and LY294002, the control and sensor cells (5 × 10^3^) were seeded into 96-well tissue culture plates and cultured in DMEM containing 2% FCS for 24 h. The cells were then treated with 10 μM, 20 μM compound suspended in dimethyl sulfoxide (DMSO) or DMSO as a control for 24 h. The concentrations of the compounds were used according to the previous studies [19,20,21].

### 2.6. Luciferase Assay

The promoter activities of the sensor cells were determined by luciferase assay as described previously [10,19,22]. The luciferase activity of the sensor cells was expressed as “Normalized luciferase activity” that was calculated by taking the luciferase activity of the control cells at 1.0.

### 2.7. Immunoblot Analysis

The cell lysates were prepared from the hGT4-0.3-sensor cells treated with 20 μM U0126 or DMSO for 24 h. Immunoblot analysis using the antibodies against p44/42 MAPK and phosphorylated p44/42 MAPK was conducted, and the band intensity was quantified as the method described previously [8,22].

### 2.8. Quantitative Real-Time Reverse Transcription-Polymerase Chain Reaction (RT-PCR) Analysis

Total RNA fractions were prepared from the SW480 cells treated with 20 μM U0126 or DMSO for 24 h. The expression levels of the β4GalT4 gene were examined by quantitative real-time RT-PCR analysis as described previously [8,10,22]. The gene-specific primers used were as follows: β4GalT4, F: 5′-GCGAAGACGATGACCTCAGACTC-3′, R: 5′-CTCCAGACTCGTGACACTTGGTGTA-3′; glyceraldehyde 3-phosphate dehydrogenase (G3PDH), F: 5′-GCACCGTCAAGGCTGAGAAC-3′, R: 5′-TGGTGAAGACGCCAGTGGA-3′.

### 2.9. Statistical Analysis

All experiments were performed three times, and the mean values with standard deviations were shown. The results of luciferase assay were analyzed with the analysis of variance followed by the Bonferroni correction for multiple comparisons. The results of quantitative real-time RT-PCR analysis were analyzed by Student’s *t*-test [10,22].

## 3. Results and Discussion

### 3.1. Establishment of Two Sensor Cell Lines with β4GalT4 Gene Promoter Regions

**Figure 1:**
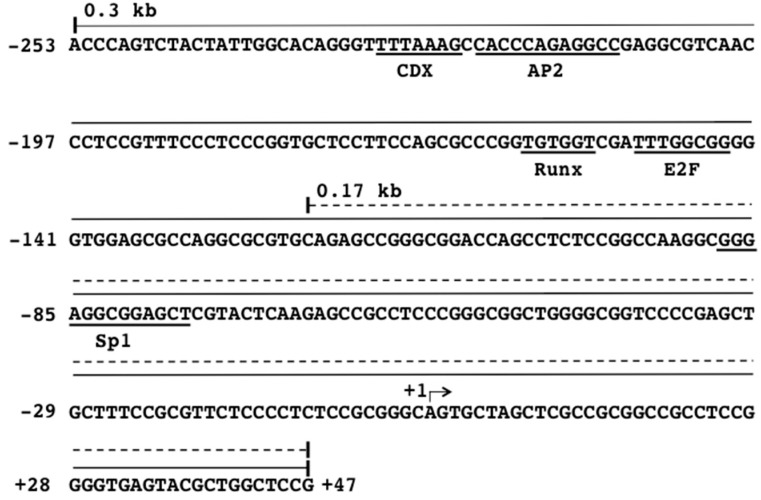
The nucleotide sequence of the human β4GalT4 gene promoter with transcription factor binding sites. The transcriptional start site is indicated with arrow. The numbers show the nucleotide positions from the transcriptional start site (+1). The reporter plasmids, pGL4-0.3 and pGL4-0.17, were prepared by insertion of the 0.3 kb and 0.17 kb promoter regions into the pGL4.15[luc2p/Hygro] vector, respectively.

Our previous study revealed that one Sp1-binding site in the 0.17 kb core promoter region is important for the expression of the β4GalT4 gene in SW480 cells [10]. Since Sp1 is well-known transcription factor involved in the regulation of the housekeeping genes [23], it may be hard to discover the drugs that specifically inhibit the expression of the β4GalT4 gene in colon cancer cells simply by using the core promoter region. In the previous study, slightly but significantly higher activities were associated with the 0.3 kb promoter than the 0.17 kb promoter [10]. When the promoter region between nucleotides −253 and −123 was analyzed by TFBIND program [24], one binding site each for activator protein 2 (AP2), E2F, caudal-related homeobox transcription factors (CDX), and Runt-related transcription factors (Runx), was found (Figure 1). These transcription factors have been shown to be closely associated with the progression and metastasis of colon cancer [25,26,27,28]. Therefore, we considered that the anti-colon cancer drugs can be discovered by the combination of two sensor cell lines having the luciferase reporters driven by the 0.17 kb and 0.3 kb promoters, and two sensor cell lines were established. 

**Figure 2:**
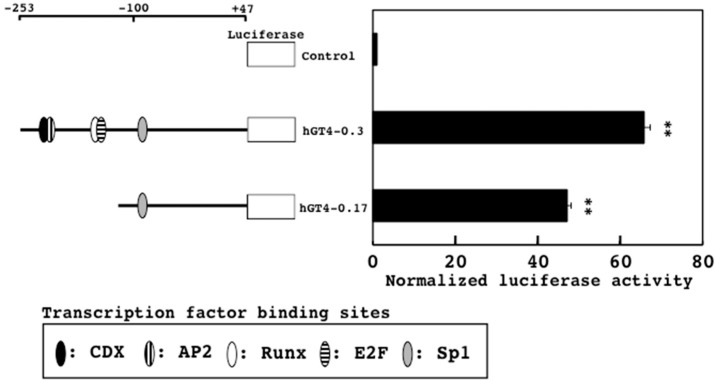
Promoter activities of the hGT4-0.3- and hGT4-0.17-sensor cells. Schematic drawing of two reporter constructs with the transcription factor binding sites are shown in the left panel. The promoter activities of the sensor cells are shown in the right panel. The luciferase activity of the control cells was set at 1.0. Data show means ± S.D. **, *p* < 0.01 against control.

The promoter activities of the hGT4-0.3- and hGT4-0.17-sensor cells showed 66- and 47-times higher than those of the control cells, respectively (Figure 2). Comparing the promoter activities among so far analyzed β4GalTs and sensor cells [10,19,22], the hGT4-0.3- and hGT4-0.17-sensor cells gave satisfactory activities. Interestingly, much higher activities were associated with the hGT4-0.3-sensor cells, suggesting that the transcription factors such as AP2, CDX, E2F, and Runx are involved in the activity of the 0.3 kb promoter cooperatively with Sp1.

### 3.2. Responsiveness of Sensor Cells to Mithramycin A

**Figure 3:**
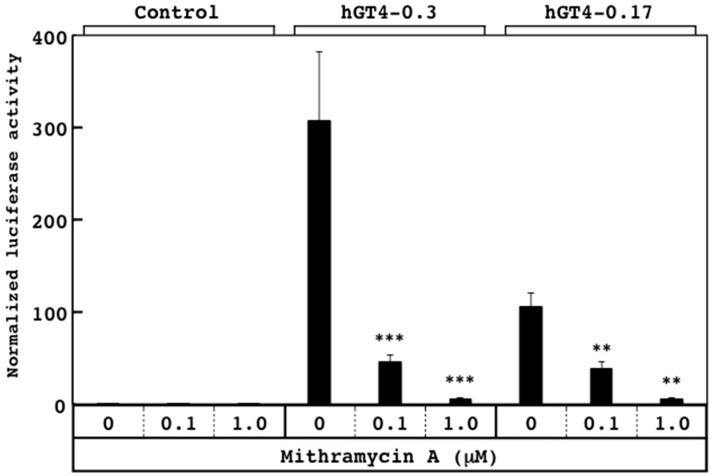
Effects of mithramycin A on the promoter activities of the hGT4-0.3- and hGT4-0.17-sensor cells. The luciferase activity of the control cells was set at 1.0. Data show means ± S.D. **, *p* < 0.01 and ***, *p* < 0.001 against control.

Both sensor cells possessed the luciferase reporters driven by the β4GalT4 gene promoters containing one Sp1-binding site (Figure 1). To examine whether or not the promoter activities reflect the effects of the compounds, the sensor cells were treated with mithramycin A, which inhibits the binding of Sp1 to its binding site, thereby suppressing the promoter activation [29]. Upon treatment with mithramycin A, the promoter activities of both sensor cell lines decreased linearly in a dose-dependent manner (Figure 3), indicating that the promoter activities of the sensor cells sensitively reflect the effects of mithramycin A. 

### 3.3. Responsiveness of Sensor Cells to U0126 and LY294002

**Figure 4:**
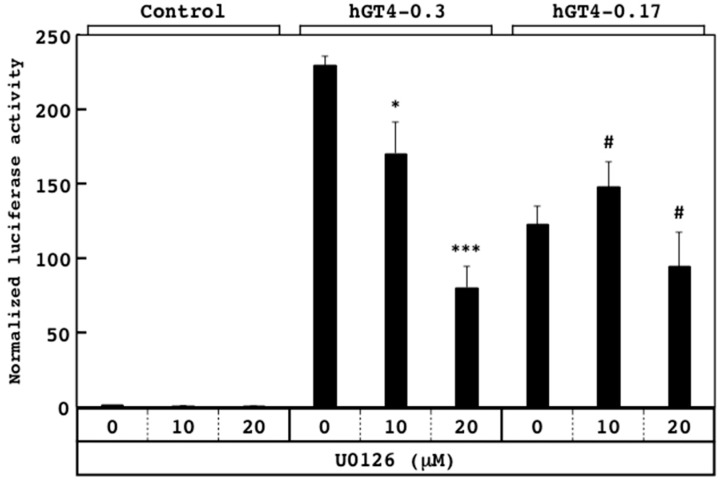
Effects of U0126 on the promoter activities of the hGT4-0.3- and hGT4-0.17-sensor cells using 96-well tissue culture plates. The luciferase activity of the control cells was set at 1.0. Data show means ± S.D. *, *p* < 0.05 and ***, *p* < 0.001 against control. #, no significant difference against control.

**Figure 5:**
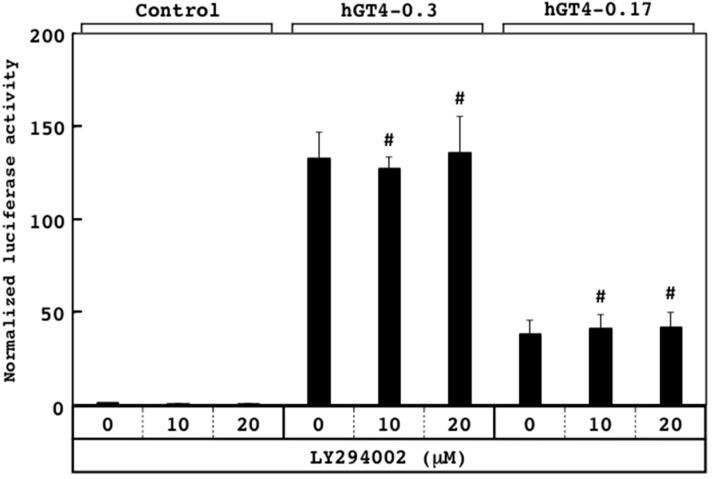
Effects of LY294002 on the promoter activities of the hGT4-0.3- and hGT4-0.17-sensor cells using 96-well tissue culture plates. The luciferase activity of the control cells was set at 1.0. Data show means ± S.D. #, no significant difference against control.

For application to high-throughput screening, the sensor cells were seeded into 96-well tissue culture plates. Since the colon cancer-related transcription factors are downstream targets of cellular signaling, and the mechanisms of action of the signal transduction inhibitors were well characterized so far, the responsiveness of the sensor cells to two inhibitors was examined. Upon treatment with MAPK kinase inhibitor U0126 [30], the promoter activities of the hGT4-0.3-sensor cells decreased significantly in a dose-dependent manner, while those of the hGT4-0.17-sensor cells unchanged (Figure 4). On the other hand, upon the treatment with LY294002, which is an inhibitor for phosphatidylinositol 3 (PI3) kinase [31], no significant responsiveness was observed for both sensor cell lines (Figure 5). These results suggest that the responsiveness to U0126 differs between two sensor cell lines, probably due to the presence of the binding sites for the different transcription factors in each promoter region.

### 3.4. Effects of U0126 on Expression of β4GalT4 Gene

**Figure 6:**
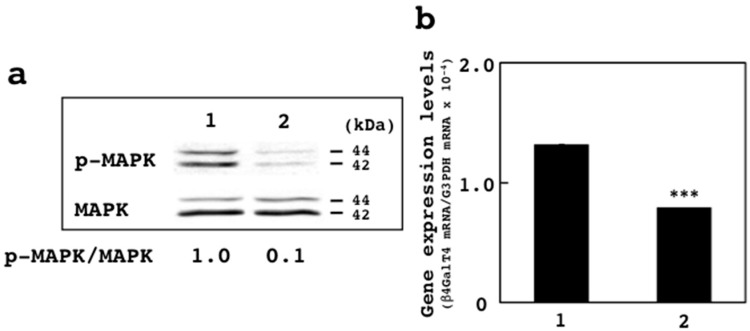
Effects of U0126 on the phosphorylation of MAPK and the expression of the β4GalT4 gene. (**a**) Immunoblot analysis of the cell lysates from the DMSO- (lane 1) and U0126-treated sensor cells (lane 2). Blotted filters were incubated with anti-p44/42 MAPK and anti-phospho-p44/42 MAPK (T202Y204) antibodies. The ratio of amounts of phosphorylated MAPK against MAPK between the DMSO- and U0126-treated cells is shown at the bottom of the blots. (**b**) Comparison of the expression levels of the β4GalT4 gene between the DMSO- (1) and U0126-treated SW480 cells (2). Data show means ± S.D. ***, *p* < 0.001 against control.

Upon treatment with 20 μM U0126, the promoter activities of the hGT4-0.3-sensor cells decreased by 65% as compared with those of the DMSO-treated cells (Figure 4). Since higher inhibitory effects were observed, whether or not the treatment with U0126 affects the expression of the β4GalT4 gene was examined. By the treatment of the hGT4-0.3-sensor cells with 20 μM U0126, the phosphorylation of p44/42 MAPK decreased dramatically when compared with the control cells (Figure 6a), indicating that the MAPK signaling is suppressed by the treatment with U0126. Under the same condition, the expression of the β4GalT4 gene decreased by 40% as compared with the control cells (Figure 6b), indicating that U0126 suppresses the expression of the β4GalT4 gene in SW480 cells. Taken together, these results demonstrated that the screening method using the sensor cells is potentially useful for the discovery of inhibitors to suppress the expression of the β4GalT4 gene in colon cancer.

### 3.5. Screening Strategy for Anti-Colon Cancer Drugs

**Figure 7:**
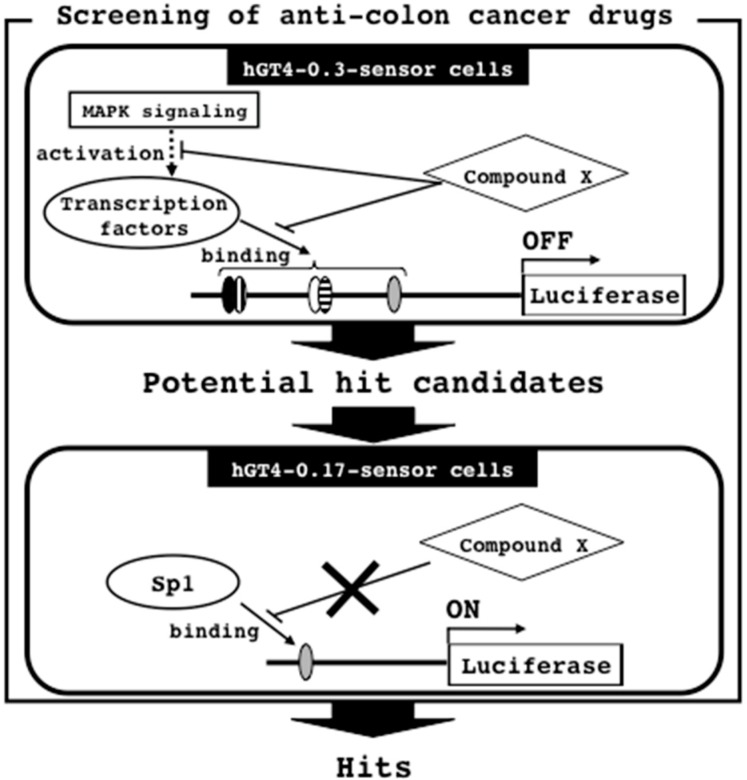
Screening strategy for anti-colon cancer drugs by the combination of two sensor cell lines. The transcription factor binding sites with symbols are the same as in Figure 2.

The present study describes the establishment of two sensor cell lines having the luciferase gene under the control of the β4GalT4 gene promoters from SW480 cells, and the potential usefulness of the sensor cells for screening of anti-colon cancer drugs. The assays were highly reproducible and showed little inter-well variability. Since the responsiveness to U0126 differs between two sensor cell lines (Figure 4), the anti-colon cancer drugs can be discovered by the combination of two sensor cell lines. Herein, we propose the screening strategy as illustrated in Figure 7. 

For the initial screening, the hGT4-0.3-sensor cells were treated with the compound library, and the potential hit candidates are selected by assessing the suppression of the promoter activation (Figure 7). These compounds are considered to inhibit the MAPK signaling pathway and the binding of transcription factors including the colon cancer-related transcription factors and Sp1 to the β4GalT4 gene promoter region. In order to exclude the possibility that the compounds inhibit the expression of the housekeeping genes regulated by Sp1, the candidates are subjected to the subsequent screening using the pGL4-0.17-sensor cells, and then the hits, which do not affect the promoter activities, are identified (Figure 7). The hits may not inhibit the binding of Sp1 to the β4GalT4 gene promoter region. After the screening, in order to show the effectiveness on the malignant properties of colon cancer cells, the cells were treated with the hits, and then subjected to the analyses such as anchorage-independent cell growth, cell migration, tumorigenic and metastatic potentials by the methods as described previously [8,22]. To avoid the resistance to chemotherapy, the anti-cancer drugs with different action mechanisms are required. Since a number of molecules are involved in the expression of the β4GalT4 gene, the proposed screening strategy is not restricted to identify the inhibitors for the β4GalT4 gene expression, and can identify the various inhibitors with different action mechanisms including the inhibitors for MAPK signaling and the binding of transcription factors.

### 3.6. MAPK Signaling and β4GalT4 Gene Expression

The MAPK signaling is known to be involved in tumor growth and progression [32]. Since the treatment with U0126, which inhibits MAPK signaling [30], suppressed the expression of the β4GalT4 gene (Figure 6b), β4GalT4 is considered to be one of the downstream targets of the MAPK signaling pathway but not the PI3 kinase signaling pathway. In colon cancer, the expression of AP2 and CDX2 has been shown to decrease [25,26], while that of E2F-1 and Runx2 has been shown to increase [27,28]. These findings suggest that the expression of the β4GalT4 gene in colon cancer is up-regulated by Sp1 cooperatively with E2F-1 and/or Rnux2 rather than AP2 and/or CDX2. The expression of E2F-1 has been shown to decrease by the treatment with MAPK kinase inhibitor PD-098059 [33]. On the other hand, upon treatment with U0126, the expression of Runx2 decreased in human thyroid carcinoma cell lines [34]. The results indicated that E2F-1 and Runx2 are downstream targets of the MAPK signaling pathway, suggesting that E2F-1 and/or Runx2 involve in the promoter activation of the β4GalT4 gene cooperatively with Sp1 in SW480 cells. In fact, E2F-1 has been shown to interact with Sp1 to regulate the promoter activation of the hamster dihydrofolate reductase gene [35]. The clinical relevance of β4GalT4 to other cancers remains to be clarified. Since the activation of both E2F-1 and Runx2 has been observed for cancers other than colon cancer, for instance, breast and pancreatic cancers [36,37,38], the expression of the β4GalT4 gene may increase in these cancers. If the increased expression of β4GalT4 is associated with poor prognosis of these cancers, the screening method will be applicable to these cancers.

## 4. Conclusions

A screening method for anti-colon cancer drugs has been established in the present study. This is the first report applying the transcriptional mechanism of the glycosyltransferase genes, which relate to the malignant potentials of cancer cells, to cell-based screening assay, and showing the potential usefulness of the sensor cells to discover the anti-colon cancer drugs, which may lead to the suppression of the malignant potentials of colon cancer by changing the cancer-related glycan glycosylation. The existence of a small portion of colon cancer stem cells is considered to be one of the causes for ineffectiveness of chemotherapy [39,40]. If the sensor stem cells are isolated from the sensor cells, the effective drugs for colon cancer stem cells could be discovered by using the sensor stem cells, which may overcome to the poor clinical outcome of colon cancer.
